# Translating stem cell research into development of cellular drugs—a perspective from manufacture of stem cell products and CMC considerations

**DOI:** 10.1111/cpr.13203

**Published:** 2022-02-14

**Authors:** Yu Alex Zhang, Glyn N. Stacey

**Affiliations:** ^1^ Zephyrm Biotechnologies Beijing China; ^2^ National Stem Cell Resource Center Chinese Academy of Sciences Beijing China

**Keywords:** cellular drugs, CMC, GMP, pluripotent stem cells

## Abstract

The development of human pluripotent stem cell (PSC)‐derived medicinal products has been gathering steam in recent years, but the translation of research protocols into GMP production remains a daunting task. The challenges not only reside with the nature of cellular therapeutics but are also rooted in the general inexperience in industry‐scale production of stem cell products. Manufacturers of PSC‐derived products should be aware of the technical nuances and take a holistic approach toward early planning and engagement with their academic partners. While not all issues will be readily resolved soon, the collective knowledge and consensus by the manufacturers and key stakeholders will help to guide rapid progression of the field.

## Abstract 2

This paper highlights and discusses the range and timing of issues that must be considered in the development of cell‐based medicines. Considerable work can be done to assure the robustness of a manufacturing system during early research and development which can then be refined more smoothly during product development. Development of characterisation and safety testing proceeds from a stage and potency assay development may continue into clinical phases.

## 1 INTRODUCTION

While much resource has been put into translational research over the last 20 years, the development of human pluripotent stem cell (hPSC)‐based clinical trials has been slow.^1^ However, the dramatic early‐stage clinical successes observed with CAR T immunotherapies have highlighted the potential benefits of new cell‐based therapies and have driven a significant stimulus in regenerative medicine in recent years. Protocols have been established for differentiation of hPSCs into many cell types of endodermal, mesodermal, and ectodermal origin and considerable effort focused on how to translate such protocols into versions suitable for development as GMP‐compliant pharmaceutical products.^1^, ^2^, ^3^, ^4^ Eighty‐seven formally regulated clinical studies based on products derived from hPSCs have recently been verified by the hPSCreg project in Europe (information kindly provided on December 6, 2021, by S Kobbold, Robert‐Koch Institute), and early successes have been reported.^5^ Encouraging news also came from the US and China as two recent IND approvals by the FDA (VX‐880 for Type 1 Diabetes^6^ and MSK‐DA01 for Parkinson's Disease^7^) and one IND by the NMPA (M021001 for meniscus injury^8^) further speed up the process of developing PSC‐derived cells into new medicine. These verified preclinical studies and clinical trials cover the development of more than 11 disease indications including product cell types with the characteristics of retinal pigmented epithelial cells, dopaminergic neurons, cardiomyocytes, and mesenchymal stromal cells.^1^, ^9^ Thus, there is an increasing likelihood that there will be novel hPSC‐based medicines appearing in the relatively near future. The next significant challenges are the establishment of the necessary manufacturing and distribution technologies to enable widespread, high volume, and cost‐effective roll‐out of these new products, with suitable safety and quality assurances to encourage their acceptability and uptake. In this article, we review some of the key generic issues in translation of research protocols and data into an Investigational New Drug (IND) application prior to clinical trials.

## 2 THE CHALLENGES OF MANUFACTURE OF CELLULAR PRODUCTS AS COMPARED TO LARGE MOLECULE BIOLOGICS

Despite advances in stem cell biology and experimental cell therapy in animal models, the industrial‐scale production of stem cell products for drug development remains a daunting task.^10^ One significant challenge is to assure optimal efficacy and safety in products which are at such an early stage of development where precise conditions to achieve optimal safety and efficacy have yet to be fully developed. In this situation, the levels of uncertainty require product‐specific development of a thorough understanding of the biological system involved and what factors can significantly affect it. Such complete understanding is unlikely to be readily achieved and additional product development time may be required, thus delaying efforts in standardization. Even then, uncertainties will remain to some degree and this is not to be unexpected for biological medicines in general. Another aspect of the challenge is to achieve sufficient cell numbers consistently, reproducibly, and economically for multiple patients. This is often highly variable between different product types and will require a good understanding of and experience with the cell culture system in question and a clearly defined product dose and anticipated immediate demand for dose numbers. Adding to the complications is that when developing appropriate standardized approaches for their manufacture, one also has to recognize that cell‐based medicines are biologically complex and highly diverse in their cellular composition and functionality.

PSCs and their differentiated products display similarity to biologics development in that the general pathway and mechanisms to suitability and regulatory acceptance are consistent, which include:

- Demonstration of the suitability of raw materials: Traceability to origin and their preparation processes should be documented and of known and ideally minimized risk or mitigated. In order to uphold the fundamental medical principle of doing no harm, evaluation of risk is primarily focused on contamination with potentially infectious agents. Other considerations that have to be addressed for raw materials include batch consistency, stability, and robustness of the supply chain.
- Consistent and sufficiently scaled‐up manufacturing process: It is an essential early step to consider the numbers of therapeutic cells required per dose for the intended number of patients to be treated from each production batch. This is key to establish the degree of scale‐up and type of culture system required.^11^ This will also be affected by the production culture's preference for adherent or suspension format and culture environment. Straightforward “scale‐out” approaches can often prove effective as a first option for cell expansion to anticipated production scales.^12^ Methods and tools (such as bioreactors) that work well at bench‐scale may be difficult to reproduce at scale particularly if they are prone to contamination or yielding variable culture batches.^13^ Thus, it is important to allow sufficient development time to demonstrate proficiency and reliability of the proposed scale‐up mechanism.
- Standardization of the product evaluation: The three pillars of biological product standardization are identity (i.e., the unique profile of features that identify the intended cell type), potency (the biological activity per dose), and purity (any unintended components are present).^14^, ^15^ It is important to recognize that markers of a particular cell type used to describe “identity” do not necessarily infer the required biological activity, and thus, the use of identity markers as a measure of potency should be avoided and would require clinical validation. Demonstration of potency is typically dependent on a functional assay, and the readout should relate to a specific biological activity. This activity is typically problematic to measure quantitatively and standardize, and the approach taken for other biologicals is to establish an arbitrary unitage based on a stable reference preparation that can be used to compare production lots. However, ideas on what reference materials might be useful for potency assessment of cell therapies are still in their infancy, although such control for performance of the analytical methods used may be feasible.

## 3 THE KEY STEPS IN MANUFACTURE OF CELL‐BASED MEDICINES

Similar to the manufacture of large molecule biologics, there are three key steps in the development of a hPSC‐based product manufacturing process, the establishment of seed stocks, the expansion/differentiation protocols, and the final formulation and storage.

Establishment of qualified seed stocks starts with the selection of appropriate hPSC lines.^2^, ^16^ Failure to secure suitable cell stocks will impact on any further development. Cells established in‐house will require investment in resources and time‐consuming qualification. Cell lines sourced from an external provider can help to significantly reduce this delay, but will require a careful due diligence process.^17^ A two‐tiered cell bank system which comprises a Master Cell Bank (MCB) and Working Cell Bank (WCB) would provide supply for continued manufacture of the cellular product. While MCB is generally made from the initial PSC stock, WCB is derived from one or more aliquots of the MCB and is used directly as the starting material for manufacturing process. While there are already existing cell banks of PSCs available worldwide,^17^ it is recommended that the drug developer should establish its own MCB and WCB so as to ensure the quality of each cell bank and of the testing performed on each bank. The ICH guideline (Q5D)^18^ and other international guidance^19^provide the general requirements for the construction of the two‐tired cell banks, whereas more attention should be paid to the appropriate characterization and testing regime that is specific to PSCs.^16^

A robust and reliable expansion and differentiation protocol casts the basis for the production of quality PSC‐derived cellular products. Protocol development should start in early translational research but will often require significant optimization and validation studies to establish a final manufacturing process that can deliver acceptably consistent product batches. Process parameters should be investigated to understand whether they are critical to product quality and what the tolerances are. Since any production of PSC‐derived products inevitably involves a multistep differentiation process, it is important to adequately control the critical parameters and add in‐process check points to ensure that differentiation is moving in the right direction. In addition, genetic and phenotypic changes can occur during the expansion phase and opportunities to check suitability of product intermediates and final product should be considered.^20^, ^21^ It is also important to verify that the bioanalytics used for cell preparations are capable of detecting any changes that would impact on the quality and safety of the product.^21^

The proper formulation and storage of final products allows the safe and reliable delivery to the patients. Further materials used as excipients that may be needed to assure product formulation stability will need to be evaluated and validated. This will include those materials added to promote sustained viability in both cryopreserved and non‐frozen cell products. The establishment of successful cryoprotectants and suitable storage may take considerable development time and that will need to be factored in.^22^, ^23^

At the point of entering clinical trials, it is crucial to have key critical manufacturing controls in place to assure the quality and safety of the production cell substrate and raw materials (reagents and consumables); in addition to which, these key controls should include in‐process control and product release.

## 4 FACILITY GMP REQUIREMENTS

Fundamental to the delivery of safe and reliable medicinal products is that the product is consistently manufactured and does not cause harm to patients. Implementation of GMP requirements supports these aims by ensuring that all materials, processes, facility operation, and staff training are documented and traceable.^24^ GMP will also ensure that validation of all of these aspects together provides a consistent final product according to standards specified by the manufacturer for quality and safety. In contrast to many other biological products like antibodies and glycoproteins, a cell culture‐based product will not be amenable to terminal sterilization. Thus, to assure avoidance of contamination and consequent infection risk in patients, it is therefore vital in translational development to consider closed systems for handling cells and maintenance of low particulate levels in the cell culture environment. Typically, any open procedures where cells could be directly exposed to the environment are expected to be performed in Grade A air (up to 3520 0.5μm particles/m^3^ at rest or in operation), whereas areas immediately outside these zones (i.e., outside cell culture biological safety cabinets or isolators) maintained at a minimum of Grade B air quality (up to 3520 0.5 m particles/m^3^ at rest and 352000 0.5 μm particles/m^3^ in operation).^25^ This general principle of “Grade A in background Grade B” requirement of the facility may be lowered only when closed systems are utilized with full Quality Risk Management (QRM) assessment. Such requirements to which routine environmental testing are added necessitate special arrangements and equipment for laboratory access, cell culture processing, and waste disposal, which can have significant impacts on the way in which culture work is performed. Furthermore, a significant source of contamination will be the operators who will be required to wear special clothing or use glove ports to handle cultures in isolator systems. Even the basic procedures of culture manipulation, microscopical observation and passaging cells become far more demanding and time‐consuming compared with the research laboratories in which the original protocols are developed. Thus, careful consideration must be given to this challenge during the translation from research protocols to GMP process, since solutions may be required which can be time‐consuming to develop and validate.

In the operation of cleanroom GMP facilities, it is also a requirement to demonstrate that an aseptic process has been maintained and this will involve routine environmental screening for particulates and microbiological contamination together with the application of process integrity tests for cell culture processes to assure they do not permit environmental contamination of the product. It is also to be noted that the use of antibiotics to ensure sterility in manufacturing processes that work well in a research laboratory may not be so robust and suitable when translated to a GMP environment.

## 5 POTENCY AND VIABILITY

Cell‐based medicines are exquisitely responsive to changing environmental conditions and understanding the most appropriate cell culture parameters (quality attributes) that will enable assurance of sustained efficacy and safety of the final product can be very challenging. Traditional viability measurements may not be linked directly to the required cell functionality, and in addition, markers classically associated with functional cells in the native tissue may not relate to the potency of a particular hPSC‐based product which is an in vitro artifact.^26^

It is crucial to establish early understanding of the intended mode of action (MOA) of the product before starting product development. In cases of cell replacement therapy (i.e., dopaminergic (DA) neurons for Parkinson's disease) where young healthy cells are to replace old dying cells of the same type, the MOA appears to be straightforward. While thorough analysis of the molecular profiling of cells can generally assure the identity of cell type, potency measurements may often require complex and prolonged in vitro and in vivo assays. DA01, for example, is a DA neuron precursor currently in phase I clinical trials with Parkinson's patients. Even though DA01 already possesses most of the molecular signatures of DA neurons, its function will have to be tested by a whole slew of electrophysiological and biochemical experiments (or even animal behavior tests) weeks to months after the cells have been cultured under maturation condition or transplanted into animal brains.^2^, ^27^ Such assay regime is obviously not suitable to be used as release tests for different production batches. It is, therefore, important to take an orthogonal approach to identify a set of parameters that would assure both identity and activity of the product.

Manufacturers may also need to consider the possibility that their product has more than one mode of action. For example, mesenchymal stromal cells (MSCs) have been reported to potentially have 9 pathways to interact with the immune system and those relevant to the particular application need to be defined and controlled.^28^ The difficulties in establishing bioassays for these activities can be extremely challenging, and in some cases, identification markers of the active cell type have been used release criteria to infer product potency. However, it is important to recognize that these are surrogates for potency which may not accurately correlate with the desired biological activity; thus, manufacturers should aim to establish bioassays for potency through the most relevant in vitro and sometimes even in vivo tests.

It is considered best practice to begin development of product characterization and development of potency assays from the earliest stages of product development.^20^ As this process advances, it is also wise to evaluate a number of alternative potency assays prior to clinical studies. At the end, any potency assays will need to bear the tests of the clinical trials. Failure to establish a clear correlation between product activity in a potency assay and the clinical benefits might further delay the development and approval of a product. Such was the case for Remestemcel‐L, whereas potency assays like TNFR1 expression was consistent with the proposed MOA of the product, it was deemed not adequate in demonstrating a clear relationship between the TNFR1 levels and clinical effectiveness.^29^ It is, therefore, an iterative process whereas the proposed potency assays will be tested and re‐tested in the preclinical animal studies and clinical trials.

## 6 THE KEY ISSUES TO BE ADDRESSED FOR DURING TECHNOLOGY TRANSFER

As PSCs have to differentiate into functional cells before they can be further utilized as medicinal products, it is more often than not that the drug development process will involve a technology transfer from an academic laboratory who invents the differentiation protocol to its industry partner. From a manufacturing perspective, it generally takes three stages from the initial due diligence to GMP production to support clinical development. (Table 1 for details). For a successful technology transfer of PSC‐derived products, it is likely that close interaction between the academic and industry partners will need to persist through all 3 stages or even beyond. Because of the innovative nature of PSC‐derived products and the lack of general industry and regulatory experience, special attention should be paid to the robustness of the protocol, measurement criteria and data format, and the reproducibility of preclinical studies.

**TABLE 1:** Key requirements in technology transfer for GMP manufacture

Stage		Contents	Technology	Purpose
1	Initial due diligence	Checklist of product information	Product description Cell line history and traceability Development reports Development data integrity	Documented evidence
		Risk identification and gap analysis	Raw materials Cell line identity and characterization Cell culture and differentiation process protocol Product characterization Equipment and facility Tacit knowledge	Technology transfer Preparation for CMC development strategy
2	Pilot studies	PSC banking, characterization, and release test	cGMP compliance ICH Q5D guideline compliance	Development of a reliable manufacturing process Establishment of release criteria
		Material attributes	Risk assessment and test	
		Process development	Cell culture and formulation process parameters optimized based on knowledge gained and DoE technology	
		Analytical methods development	Cell variability, identity, purity, microbial agents, and potency profile	
3	Manufacturing for development	Manufacturing site validation	cGMP compliance	Confirmation of process robustness and reproducibility Control strategy and product specification Support of product expiry date
		Material control	Vendor audit Quality control	
		Analytical methods validation and test	Batch analysis and in‐process control	
		Manufacturing and process characterization	Process validation, acceptable range of operation	
		Product stability testing	Long‐term and accelerated sample test	

In order to assure a reliable manufacturing process that delivers a bulk product that is consistent in its quality, safety, and timing of deliver, it is important to understand the key variables that could impact on these parameters. However, it is often said that “cell culture is an art!” and the variations observed in cell cultures can mean that bulk product can need quite wide acceptability criteria and suffer from high batch rejection rates due to contamination or variation in properties and time required to achieve acceptable product.

The measurement of cell systems has historically been problematic due to the use of evaluation methods based on qualitative values and operator interpretation. Ill‐defined parameters such as passage number, microscopic morphology, confluency, etc., can yield culture processes that deliver completely different outcomes in different laboratories or even between different workers. In addition, the use of parameters that are not directly related to function can be highly misleading and data that gives and average datum for all the cells in a culture can hide significant cell culture heterogeneity and thus may not give a clear picture of the reproducibility of quality between batches. Compounding issues are the common practices and data format utilized in an academic setting, such as paying attention to improvements of key ingredients and achievements of desired cellular phenotypes, yet neglecting or missing proper controls of the environment and equipment, lacking any in‐process controls. This can sometimes cause difficulty in the interpretation of the final results. A key series of decisions that need to be made in preparing cell metrics should first address selection of the most informative parameters, secondly, selecting appropriate methods to give a reliable and reproducible result, and thirdly, setting realistic tolerances that both enable useful product and do not result in unnecessary waste of batches with acceptable functionality.

Preclinical studies although not directly impacting on patient safety will be used by manufacturers to help demonstrate quality and safety of the manufacturer's process to regulators. Preclinical studies performed during early stages of product development (i.e., prior to manufacturing protocol is finalized) tend to be informative, and sometimes even critical in decision making, they are nonetheless not fully representative of the final product going into the clinics. On the contrary, repeating every single preclinical study using the products from the final production batches will be costly, time‐consuming and may be unnecessary. Thus, it is important to have a holistic approach toward preclinical study design, such as the cellular products from representative batches are fully compared and the reproducibility of preclinical results from different batches are ensured.

## 7 CONCLUSION AND RECOMMENDATIONS FOR NEWCOMERS TO PRODUCT DEVELOPMENT

As PSC‐derived medicines are entering mainstream drug development, new opportunities as well as challenges arise for manufacturers of the cell products. While there are still ample uncertainties regarding their industry‐scale production, research in the past 20 plus years has provided a basis for safe and efficacious products. Manufacturers’ experience will obviously grow and mature in the coming years with the support from the collective knowledge and consensus of scientific, industry, and regulatory experts. For newcomers to product development of PSC‐derived medicine, it is essential to have an overall plan at the beginning and an early engagement with key stakeholders in all key issues discussed above. When carrying out due diligence on an intended product, utilizing hands‐on experience is crucial to assess the translatability of research‐based protocols to GMP manufacturing and suitability of relevant bioanalytical methods. Furthermore, it is vital to plan out all key validation experiments to ascertain reliability (cell lines and protocols) and to initiate engagement with vendors and interactions with regulatory authorities at the earliest stage as possible. Finally, it is our hope that the early developers of PSC‐derived medicine would create an open environment for the disseminating of information and building of consensus.

## CONFLICT OF INTEREST

Y.A.Z is a co‐founder of Zehpyrm Biotechnologies, which engages in the development of PSC‐derived therapeutics. G.N.S has no competing interests.

## AUTHOR CONTRIBUTIONS

This article originates from a presentation by Y.A.Z. at the 2021 PSConf (April, 2021). Y.A.Z. and G.N.S. co‐wrote the manuscript.

## Data Availability

Data sharing is not applicable to this article as no datasets were generated or analysed during the current study.
